# Finding the signal in the noise: Could social media be utilized for early hospital notification of multiple casualty events?

**DOI:** 10.1371/journal.pone.0186118

**Published:** 2017-10-05

**Authors:** Rachael A. Callcut, Sara Moore, Glenn Wakam, Alan E. Hubbard, Mitchell J. Cohen

**Affiliations:** 1 Department of Surgery, University of California San Francisco, San Francisco, California, United States of America; 2 Department of Biostatistics, University of California, Berkeley, California, United States of America; 3 Department of Surgery, University of Michigan, Ann Arbor, Michigan, United States of America; 4 Department of Surgery, University of Colorado & Denver Health, Denver, Colorado, United States of America; University of Kansas Medical Center, UNITED STATES

## Abstract

**Introduction:**

Delayed notification and lack of early information hinder timely hospital based activations in large scale multiple casualty events. We hypothesized that Twitter real-time data would produce a unique and reproducible signal within minutes of multiple casualty events and we investigated the timing of the signal compared with other hospital disaster notification mechanisms.

**Methods:**

Using disaster specific search terms, all relevant tweets from the event to 7 days post-event were analyzed for 5 recent US based multiple casualty events (Boston Bombing [BB], SF Plane Crash [SF], Napa Earthquake [NE], Sandy Hook [SH], and Marysville Shooting [MV]). Quantitative and qualitative analysis of tweet utilization were compared across events.

**Results:**

Over 3.8 million tweets were analyzed (SH 1.8 m, BB 1.1m, SF 430k, MV 250k, NE 205k). Peak tweets per min ranged from 209–3326. The mean followers per tweeter ranged from 3382–9992 across events. Retweets were tweeted a mean of 82–564 times per event. Tweets occurred very rapidly for all events (<2 mins) and represented 1% of the total event specific tweets in a median of 13 minutes of the first 911 calls. A 200 tweets/min threshold was reached fastest with NE (2 min), BB (7 min), and SF (18 mins). If this threshold was utilized as a signaling mechanism to place local hospitals on standby for possible large scale events, in all case studies, this signal would have preceded patient arrival. Importantly, this threshold for signaling would also have preceded traditional disaster notification mechanisms in SF, NE, and simultaneous with BB and MV.

**Conclusions:**

Social media data has demonstrated that this mechanism is a powerful, predictable, and potentially important resource for optimizing disaster response. Further investigated is warranted to assess the utility of prospective signally thresholds for hospital based activation.

## Background

Social media plays an ever increasing role in our society with 68% of Americans using social media including approximately 78 million persons using it multiple times per day [1,2]. Social media has played an integral role in several prior large scale disasters including the the 2011 Egyptian uprising [3], Taiwan typhoon in 2009 [4], and in the 2010 Haiti earthquake. In each of these cases, traditional communication methods were inadequate and the use of social media improved coordination efforts of disaster response and recovery. Similarly, social media has been successfully utilized in other fields to predict and respond to public health events.

Advocates believe that social media tools have great promise for enhancing our current disaster management communication strategies but studies are lacking [3]. These concepts have remained largely theoretical and the limited prior data available have focused on the descriptions of the public use of social media following various worldwide crises. Frequently, delayed notification and lack of early information has hindered timely hospital based activations in large scale multiple casualty events. Although many institutions and federal agencies have incorporated a social media response to deliver information to the public following an event [3], there has been no focused research on leveraging this platform to develop improved and early disaster notification mechanisms to law enforcement, prehospital providers, or hospital systems. Twitter, a form of microblogging, which allows two-way communication has been used to tract non-disaster public health events, and we hypothesized that it could be used to develop a prospective multiple casualty warning signal for hospital based activation. The objective of this study was to 1) initiate the development of a qualitative model designed to provide an additional tool for hospital level disaster notification and 2) to assure that the model would not falsely activate in a control event.

## Methods

### Historical retrospective tweet data

Tweets relating to five recent U.S. multiple casualty events were acquired post hoc from Twitter via Gnip's Historical PowerTrack [5]. All data was purchased from the Twitter Corporation. The tweet content including the text of the tweet, user information, geocoding of the tweet, structure of the tweet, number of each user’s followers, retweet counts, and timing of the tweets were supplied. The 5 multiple casualty events of interest included the Boston Bombing (BB), San Francisco Asiana 214 Plane Crash (SF), Napa Earthquake (NE), Sandy Hook Elementary School shooting (SH), and Marysville School Shooting (MV]) Relevant tweets posted on the day of the event and in the six days thereafter (for seven days total per event) were identified using the incident time of each event as: the Sandy Hook Elementary School shooting on December 14, 2012, at 9:35:00 EST (time of first 911 calls [6]); the Boston Marathon bombing on April 15, 2013, at 14:49:00 EDT (detonation time of first bomb [7]); the Asiana Airlines crash at San Francisco International Airport on July 6, 2013, at 11:28:00 PDT (seawall impact time [8]); the South Napa earthquake on August 24, 2014, at 03:20:44 PDT (earthquake origin time [9]), and the Marysville High School shooting on October 24, 2014, at 10:39:00 PDT (time of first 911 call [10]).

Tweets were identified using disaster-specific filtering rules (Table 1). Where more than one text pattern was listed for a given event, a tweet was included if it matched on either (or both). Filtering was performed via a case-insensitive keyword search where individual keywords were separated by boolean operators. A space denotes an 'and' operator, an uppercase 'OR' denotes an 'or' operator, and this logic is combined using parentheses to group clauses. Quotation marks indicate terms which should be matched exactly (not tokenized) [11]. Tweet data was packaged as a collection of compressed JSON files, each containing tweets from one 10 minute span of a single event's seven day collection period. Data within these JSON files was imported and 'cleaned' using the statistical software R [12] with package jsonlite [13] and subsequently summarized and visualized using package ggplot2 [14]. Any tweets posted prior to their respective event's time were excluded from the analytic sample.

**Table 1:** Search terms for 5 multiple casualty events.

Disaster Event	Search Terms
**Sandy Hook Elementary School shooting (SH)**	School (gunman OR 'Sandy Hook' OR victims OR shooting OR Newtown)
	Newtown (shooting OR gunman OR student OR Sandy Hook OR elementary)
**Boston Marathon bombing (BB)**	Bomb (Boston OR marathon OR explosion OR terrorist OR finish line)
	Boston (explosion OR terrorist)
**Asiana Airlines crash (SF)**	Plane (crash OR SFO OR runway OR San Francisco OR Asiana OR '214')
	Asiana (Crash OR '214' OR Runway OR SFO OR San Francisco)
**South Napa earthquake (NE)**	Earthquake (San Francisco OR 'sf' OR Napa OR '6.0')
**Marysville Pilchuck High School shooting (MV)**	School (gunman OR Marysville OR victims OR shooting OR Washington)
	Marysville (shooting OR gunman OR student)

Quantitative and qualitative analysis were performed to identify similarities across events and the timing of differing tweet per minute thresholds were compared to the county disaster signal for each event. Numerical summaries of interest were computed, including mean followers per user, proportion of collected tweets that were retweets, and maximum tweet-per-minute volume. To better structure visualizations for qualitative identification of common event 'signatures,' tweets meeting all criteria described above were summarized into tweets per minute during the first twelve hours after the event onset. The speed with which tweet volume reached a threshold and/or peak was compared to the timing of the traditional county disaster signal for each event. For multiple casualty events that require the resources beyond normal functioning, county governments in conjunction with local official coordinate disaster wide responses involving multiple jurisdictions and hospital systems. Each of the events utilized in this report generated a detailed after action report that produced by the responsible governing body. These reports can include detailed information on timing of the initial event, first EMS calls, hospital standby notification, and county wide disaster activation (if activated). Not all ‘after action’ reports report all metrics. These after action reports are considered the official documentation of the time line of the events and therefore, we utilized the metrics available in each report.To compare the speed with which tweets accumulated across events, the cumulative tweet volume in the first 60 minutes after each event's onset was also computed.

### Prospective test of signal thresholds

A prospective API (R-based application program interface) was then constructed using generic search terms for potential multiple casualty events and was prospectively used to gather data from Twitter via their API (application programming interface) during a recent high profile sporting event (SF Super Bowl 50, SB50). SB50 was to be used as a control event to test the tweet per minute thresholds developed from the prior 5 US multiple casualty events. We compared the multiple casualty event results to the control event to determine how often a warning would have fired in error for various tweet/minute threshold signals using potential multiple casualty search terms or ‘event’ and a ‘location’ key word (Table 2). The key words were developed by studying both the most frequently used hashtags and keywords common across the five previously analyzed events ("breakingnews", "cnn", "pray", etc.) and by utilizing knowledge of locally relevant hashtags and keywords ("sf", "sfgh", "zfgh", "thegeneral", etc.).

**Table 2:** Event & location key words utilized in the prospective test signal for Super Bowl 50.

Super Bowl 50	Search Terms
**Event Key Words**	breaking, news, breakingnews, cnn, pray, crash, shot, shooting, stab, stabbed, stabbing, fall, dead, died, accident, earthquake, flood, victim, victims, fatality, fatalities, attack
**Location Key Words**	sf, sanfrancisco, san, francisco, sanfran, prayforsanfrancisco, bayarea, sfbayarea, bay, sanfranciscobay, northerncalifornia, norcal, california, ca, sfgh, zfgh, thegeneral, general, goldengatebridge, goldengate, bridge, baybridge, bart, caltrain, muni, cablecar

The volume of Super Bowl tweets returned at any moment in time were subject to a "streaming cap": the free public filtered streams offered by Twitter "max out" at a small percentage (approximately 1%) of the total volume of tweets coming through Twitter's "firehose." When the streaming cap is exceeded, a rate limiting message is issued by the streaming service indicating how many tweets were missed. Tweets were collected into JSON files which were rotated approximately every half hour. JSON files were preprocessed via shell script and imported and cleaned utilizing the jsonlite and tidyjson [15] packages in R. Tweets containing at least one event-related keyword AND at least one location-related keyword were summarized as tweets per minute over the entire collection period.

## Results

For the 5 recent U.S. multiple casualty events, over 3.8 million study tweets were analyzed including a per-event tweet count of SH: 1,815,751; BB: 1,147,793; SF: 430,616; NE: 205,073; and MV: 249,847. The distribution of tweets included unique tweets in 45% of postings and the remaining 55% were retweets or reposting of prior tweets. Retweets were tweeted a mean of 82–564 times per event. The mean followers of the person originating a tweet were ranged from 3382–9992 across events.

**Fig 1:**
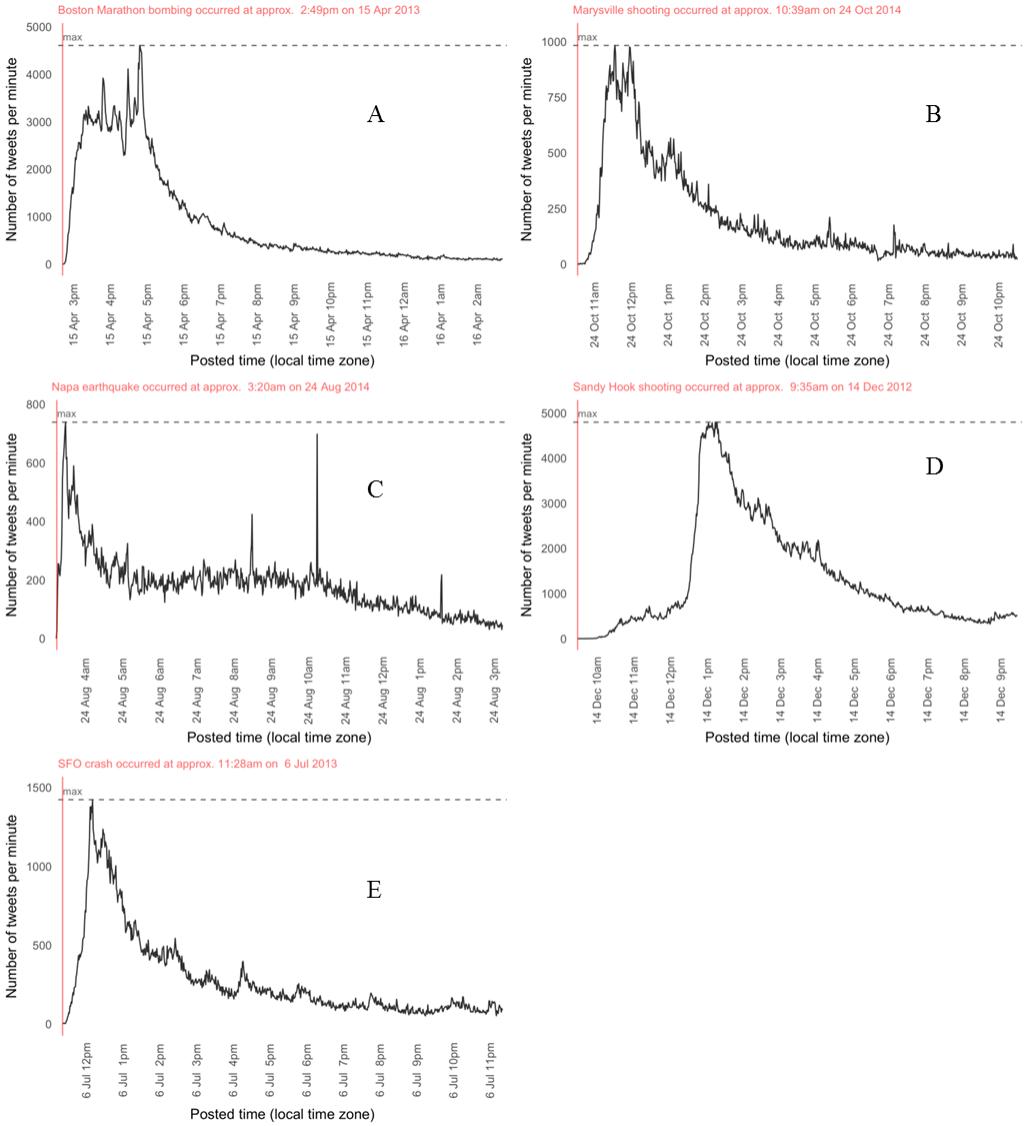
Tweets per minute in the first 12 hours following each event. Panel A: Boston Bombing; Panel B: Maryville School Shooting; Panel C: Napa Earthquake; D: Sandy Hook Elementary School Shooting; Panel E: San Francisco Air Plane Crash.

All events reached their overall maximum peak tweet posting per minute rate within 3 hours following the incident with the peak rate being reached fastest in the NE, SF, and MV (Fig 1). The tweet graphic signatures were consistent across disasters except SH which had similar signature but, delayed signal initiation (Fig 1). Peak tweets per minute ranged from 209–3326 for each event with the median in the first 60 minutes from 10–2169 (BB: 2564; SF 549; MV 162; NE 368; SH 10). The total tweets in the first 60 minutes represented a high percentage of the total tweets in the 7 days following the incident time in all cases except SH (11.5% BB, 7.8% SF, 8.0% MV, 11.9% MV, 0.14% SH).

### Identifying the signal

**Fig 2:**
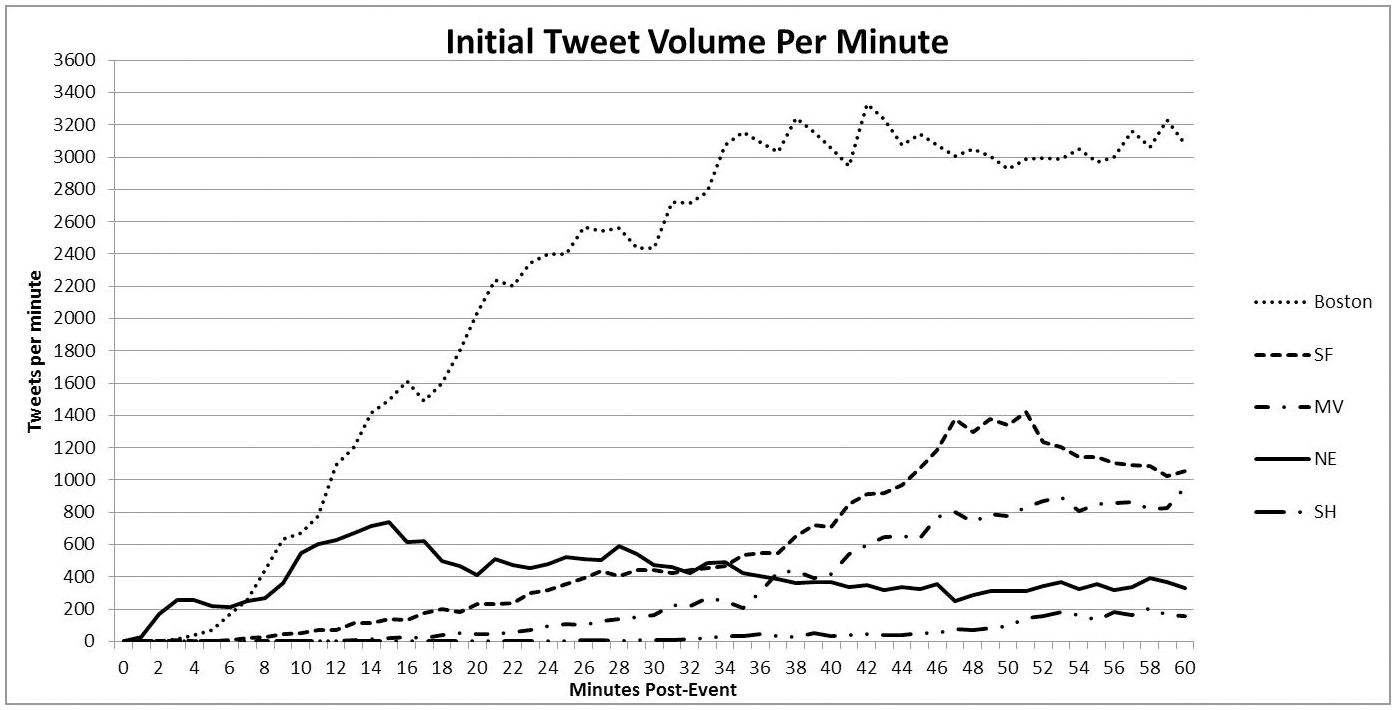
Tweet volume per minute in the first 60 minutes following the official event time. BB: Boston Bombing; SF: San Francisco Air Plane Crash; NE: Napa Earthquake; MV: Maryville School Shooting; SH: Sandy Hook Elementary School.

Initial tweets occurred very rapidly in all events with the first signals detected less than 2 mins after the incident start times. When isolating the analysis to only tweets in the first 60 minutes, 1% of the total event specific tweets were reached in a median of 13 minutes of the first 911 calls (Table 3) with the most rapid 1% threshold being reached in the NE (2 minutes), BB (9 minutes), and SF (13 minutes) (Table 3). The maximum tweets per minute in the first 60 minutes was highest for the BB (3326 tweets/minute), followed by SF (1423), MV (957), NE (739) and SH (209) (Fig 2).

**Table 3:** Tweet thresholds in the first 60 minutes following each event.

% of total tweets in first 60 minutes	Minutes Post-Event					
	BB	SF	NE	MV	SH	Median
**1%**	9	13	2	19	21	13
**2%**	11	15	4	23	27	15
**5%**	14	21	6	28	33	21
**10%**	18	26	10	33	37	26
**50%**	39	46	27	48	53	46
**75%**	49	53	42	54	57	53
**90%**	56	57	53	58	59	57
**100%**	60	60	60	60	60	60

BB: Boston Bombing; SF: San Francisco Airplane Crash, Asiana 214; NE: Napa County Earthquake; SH: Sandy Hook Elementary Shooting

Differing thresholds were compared to the county disaster notification time (if a county disaster page was activated), hospital stand by notification times (if no county disaster page was performed), and patient arrival times (obtained from after action reports). A 200 tweet per minute threshold was reached fastest with NE (2 min), BB (7 min), and SF (18 mins). If this threshold was utilized as a signaling mechanism to place local hospitals on standby for possible large scale events, excluding SH, the signal would have preceded patient arrival to local hospitals in all cases. It would also have preceded traditional county disaster notification and hospital standby notices in SF and NE, and followed within minutes in BB (2.5 minutes).

In both school shootings, a clear county wide disaster activation time was not reported in the after action reports. In the case of MV, there was an all-county wide law enforcement communication sent 28 minutes after the incident. Using this as an indicator in place of an official disaster mass casualty warning, our signal threshold would have been initiated within 2 minutes of this notice and 16 minutes prior to patient arrival at the hospital. For the Sandy Hook (SH) school shooting time line there was no clear time for the county disaster page available or hospital stand by times available in official after action reports making analysis of our signal not possible. There were also no victims transported to hospitals and the first report of casualties for SH did not occur until approximately 40 minutes after the event incident time [6].

### Testing the multiple casualty signal threshold: Super Bowl 50 case study

As a proof of concept exercise, a prospective API using generic search terms for the event (Table 2) was built in anticipation of the Super Bowl 50 to test the threshold of 200 tweets per minute for triggering notification of multiple casualty events. The goal was to test whether a high profile, frequently tweeted public event would falsely trigger the signal even if no mass casualty situation arose during the event. The API gathered relevant tweets that included one or more of the generic terms plus a location or event term for a two week period beginning one week before the Super Bowl and continuing for 7 days following the Super Bowl. An estimated 55,355,870 original tweets, retweets, and quote tweets were collected over the approximately two week period. Not included in this total were 248 partial or truncated tweets which were discarded for incomplete data. The contents of an estimated 6,675,076 additional tweets were not captured because, at some moments, the provided keywords matched more tweets than Twitter's imposed rate limit allowed to be delivered.

**Fig 3:**
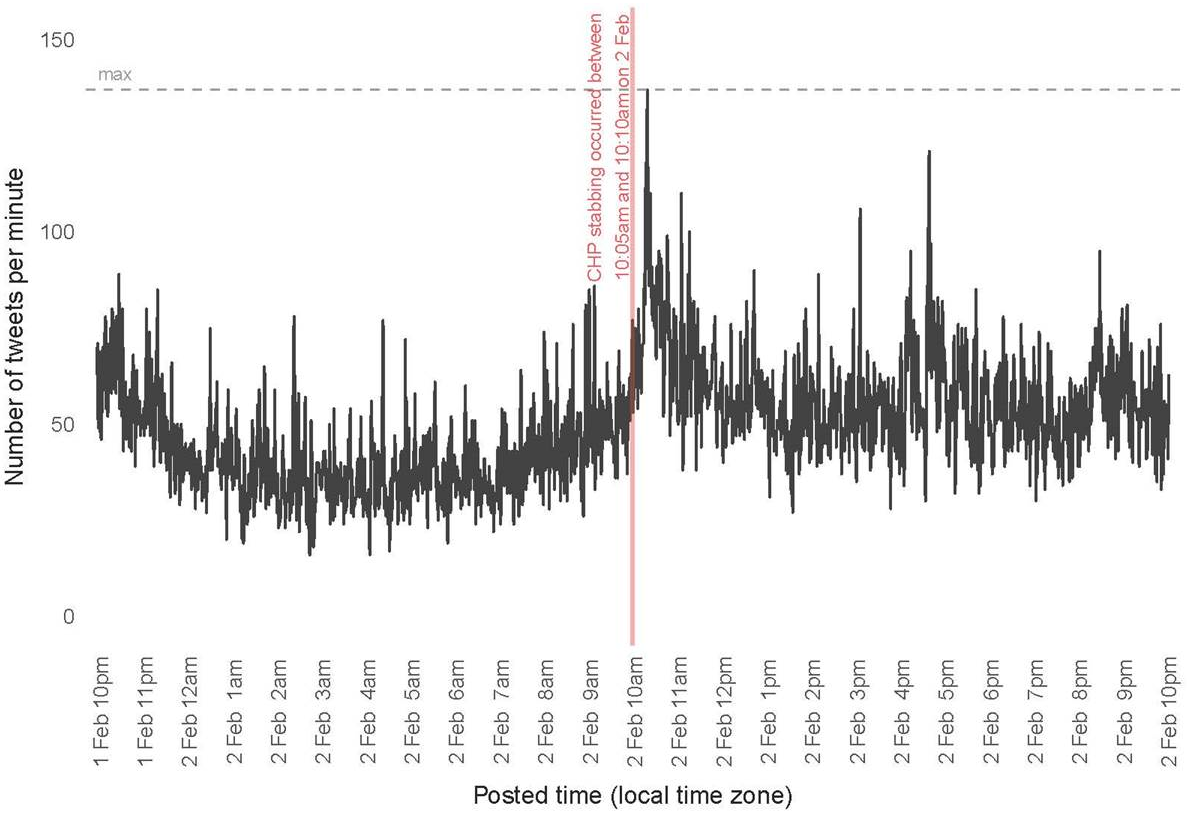
Tweets per minute during a high profile, low victim event during Super Bowl 50.

**Fig 4:**
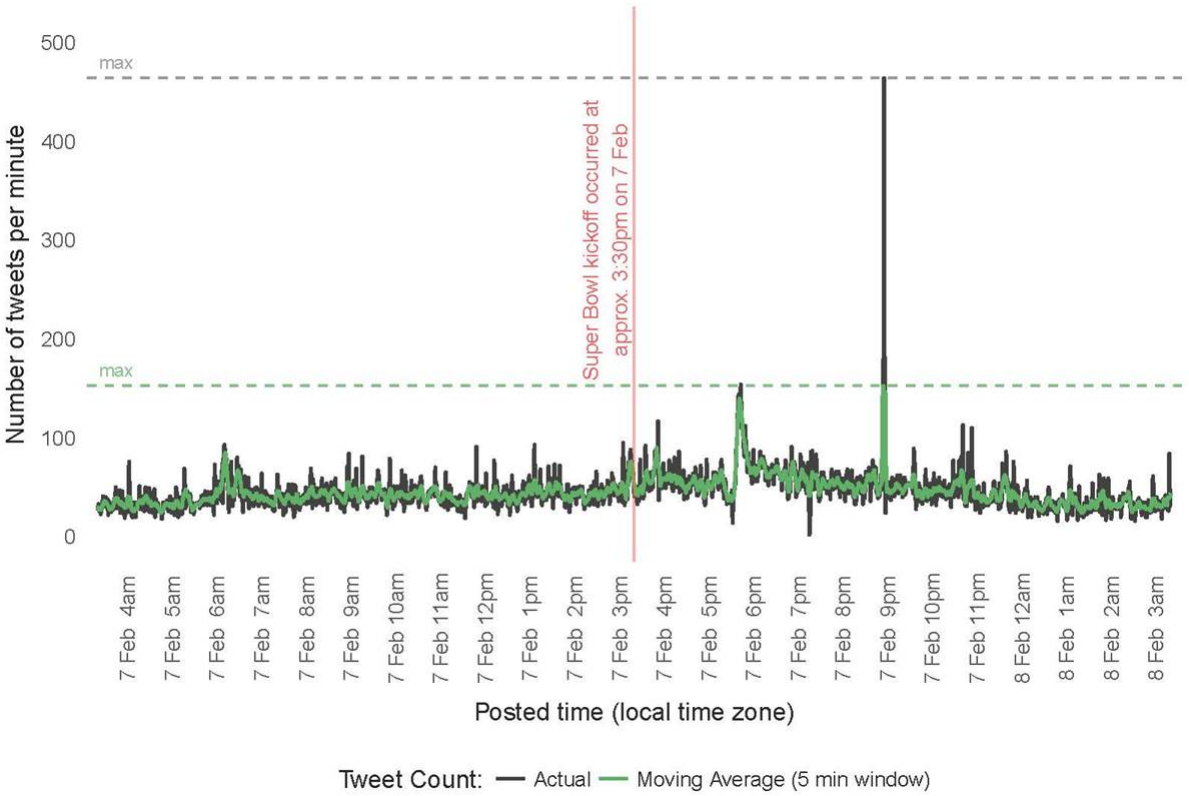
Tweets per minute during the start of the Super Bowl 50 game.

Although not planned, while the API was running, a high profile but low casualty count (one victim) event occurred during the Super Bowl near the Fan Zone Experience in San Francisco. This event provided an additional test to our signal to determine if a low casualty count but high profile trauma would falsely activate our signal. On Tuesday, February 2, 2016, between approximately 10:05 (time encounter began) and 10:10 (time of distress call) PST, a California Highway Patrol (CHP) officer was stabbed in San Francisco [16]. The first tweet about the stabbing from an SFPD officer appeared at 10:30 PST [17]. Between 10:00 and 12:00 on this date, 343,084 tweets were collected (Fig 3). After filtering the text of tweets for those which contained at least one event-related keyword and at least one location-related keyword in the text of the tweet, the total number of tweets over the two hour period was reduced to 72,952. In the two hours following this event, a signal using our key generic words with restriction to location set at the 200 tweet per minute threshold would not have fired (Fig 3) indicating that use of this as a large scale multiple casualty signal for placing hospitals on standby would have worked correctly during a high profile, low casualty event. In addition, the signal would not have been activated even during the height of the Super Bowl activities (during game time) again supported it as a potential signaling threshold for early warning detection (Fig 4).

## Discussion

Social media platforms including Twitter have become ubiquitous in our lives. It is estimated that over 68% of U.S. persons are a user of at least one form of social media [1, 2, 18]. Each minute it is estimated that 278,000 tweets are sent in the U.S. Social media is a highly interactive platform which is web based, allows two way communications between users, and occurs in a virtual space [4]. Twitter has also been described as a form of ‘microblogging’ that allows ‘dissemination of information that is timely and vast^2^’ and it has become a highly utilized format by the public following worldwide mass casualty events. Several prior case studies have been published detailing experiences with the platform ranging from directing rescuers to victims following typhoons, hurricanes, earthquakes, and terrorist events, to using the platform to exchange information about how to reach disaster response resources for recovery [4,19]. To date, research that has been conducted on using these powerful tools during times of disaster are limited to epidemiologic reports of basic Twitter use statistics and characteristics of users.

Communications in times of mass casualty events, especially those involving natural disasters, have always posed challenges. In nearly every after action report, communication is cited as the most common entity needing improvements [4, 19]. In fact, Rodriguez et al. note that ‘effective disaster communication may prevent a disaster or lessen its impact, whereas ineffective disaster communication may cause a disaster or make its effects worse [20].” As an example, in the SF Asiana airplane crash, the vast majority of the early information from the event was erroneous [21]. ‘Disaster myths’ are harmful and they can alter the way a community responds to an event [22, 23]. Although people do not intend to mislead, disasters are emotional making perception of facts often skewed [23,24].

The difficulty for hospital based providers in the age of widespread availability of social media is trying to wade through the enormous amount of information that flows almost instantaneously following these events. This information nearly always precedes the activation of any formal notification process and we believe that social media as a form of non-traditional data has great potential to provide an opportunity to improve early warning of impending need to respond to mass or multiple casualty events. As an example, the county wide disaster page activation occurred nearly 30 minutes later then information appeared on social media feeds following the crash of a commercial jet liner in San Francisco with over 300 potentially injured occupants [21]. In fact, the first Twitter posting of a plane down occurred 30 seconds after the crash and preceded even the 911 calls [21]. Drawing on the experience from the Asiana plane crash, we recognized the potential power of this modality for earlier hospital notification. Building on this conceptual idea, the present study is the first ever investigation of a prospective signally disaster preparedness activation mechanism built from these non-traditional data sources targeted at the hospital level.

Our findings confirm that information flows via Twitter extremely rapidly after an event of interest. In the 5 case events examined, the first relevant tweets appeared in under 2 minutes from the estimated start time of each event in the after action reports. These tweets were posted prior to 911 calls in all the events except for the Sandy Hook (SH) school shooting. Fig 1 demonstrates the rapid use and scalability and underscores the potential power of this modality for information dissemination. We believe that SH may have had a delayed scalability in the first hour following the event because this event took place at an elementary school where the number of persons on site during the time of the initial event with access to a device allowing posting to social media would have been extremely limited. In addition, the tweet signature is also likely a reflection of the number of secondary witnesses or the public nature of the disaster taking place. For those events occurring in highly populated areas or events, the number of potential witnesses and Twitter users will be greater than in events occurring in geographically isolated areas or low populated events.

This proof of concept study has shown that a non-traditional data source like Twitter could be utilized to develop early warning signals of multiple casualty events that require hospital based responses that exceed normal operations. A 200 tweet per minute threshold signal in this study would have resulted in hospital notification before patient arrival in all cases where injuries requiring hospital based care were present. In addition, it would have sent notification prior to or within 3 minutes of county disaster signals in cases where county wide alerts were activated. Simultaneous or nearly simultaneous notification is still valuable given that redundant communications are needed given the high rate of failure of infrastructure that often occurs following many mass casualty events.

Although more work is needed to identify the optimum tweet per minute threshold that would result in an appropriate sensitivity and specificity for a wide spectrum of hospital based disaster activations, a signal developed from generic trauma search terms coupled with terms that are geographically restricted has real promise. To be generalizable, our data utilized generic terms such as ‘crash, accident, shot, shooting, stab, stabbed, stabbing, fall, dead, died, earthquake, flood, hurricane, tornado, victim, victims, fatality, fatalities, attack’ coupled with a geographic restriction. However, to reflect the evolution of multiple casualty events since the original 5 included in this study occurred, we suggest expanding these generic terms to include ‘terrorist, and terror.’ It will be necessary if these signals are incorporated into practice, to continue to iterate on the most relevant terms as the safety and security of our world evolves.

While promising in concept, the data presented in this paper can not be used to determine the false negative rate of a signal applied across a spectrum of disaster scenarios. Disasters remain unpredictable in frequency making prospective testing of the signal challenging both from a data storage standpoint and practicality standpoint. These signals will be most helpful if they are geographically restricted, but predicting in advance that an event will definitely occur to allow testing of the adapted signal for a given region is nearly impossible. One could create a signal for a given location and no event ever takes place. In contrast, it is impractical without massive compute infrastructure to prospectively monitor all geographic areas in the world for the next large scale disaster. Thus, the determination of the false negative rate (failure to activate for a real event) will remain reliant on retrospective tweet data ascertained following future disaster events of varying types.

Although there are no standard approaches adopted widespread for the use of social media platforms for disaster planning and notification, there are many potential advantages to social media over traditional resources for communication of initial information in disaster times. Social media is inexpensive, rapidly disseminated, allows 2 way communication, scalable through ‘retweets,’ and not dependent upon a traditional power grid [25]. In fact, there have been several worldwide incidences that have cited Twitter as the only source of communication available when other traditional communications have failed [3,4]. In contrast, traditional media sources filter the message, are only 1 way communication, have a slower dissemination, lack the same scalability, are dependent upon the power grid, and exclude geographically remote areas [25]. Further, standard communication systems have failed in a number of recent events including the Taiwan typhoon in 2009 [4], 2010 Haiti earthquake [3], and alternative communication sources including Twitter were relied upon heavily in the 2011 Egyptian uprising [3]. Merchant et al. note that social media has great potential to increase ‘our ability to prepare for, respond to, and recover from events that threaten the public’s health [3].’

These social media tools coupled with advanced analytics have great promise for further development of prospective early warning disaster signals, but they also present a number of limitations. To be most effective, the signals need to be geographically specific and thus, would require some customization. In contrast, the search terms the signals are built from as demonstrated by our Super Bowl test of the signal can be useful even if they are quite generic. Events that occur in more remote geographic areas that lack access to internet would be less likely to be sensitive to a signal built on social media activity. Further, there is differential use of social media across differing age groups, socioeconomic statuses, and geographically [4]. Finally, infrastructure disruption in wide spread disasters may limit the use of social media and responding agencies must have access to social media for this tool to be effective. Each of these could limit the applicability of these types of warnings.

## Conclusion

Social media platforms including Twitter have great potential for use in formalized disaster planning and response. Social media data has demonstrated that this mechanism is a powerful, predictable, and potentially important resource for optimizing disaster response. Further investigated is warranted to assess the utility of prospective signally thresholds for hospital based activation.
